# Classroom acoustics as a consideration for inclusive education in South Africa

**DOI:** 10.4102/sajcd.v64i1.550

**Published:** 2017-09-08

**Authors:** Coralie van Reenen, Catherine Karusseit

**Affiliations:** 1 Council for Scientific and Industrial Research (CSIR), South Africa; 2 Department of Architecture, University of Pretoria, South Africa

## Abstract

**Background:**

It can hardly be disputed that a school environment should be conducive or, at the very least, not prohibitive to effective learning. The provision of fair, equal and barrier-free access to education is referred to as inclusive education. South Africa supports a policy of inclusive schooling, striving to accommodate all children, including those with disabilities, in mainstream schools. This article sets out to prove that noise control in classrooms is a relevant, yet neglected, aspect of inclusive classroom design in South Africa and requires specific attention.

**Objectives:**

The objectives of this study are to: (1) establish the impact that noise has on learners with sensory, language or learning impairments; (2) establish the preferred listening conditions for these learners by examining prior research and guidelines available in other countries; and (3) outline the current South African regulations pertaining to classroom acoustics and assess them against the preferred listening environment.

**Method:**

This research was conducted as a systematic review with reference to the South African context. Local and international research and guidelines were used as references, providing an overview and evaluation of data concerning noise and learning.

**Results:**

Noise is disadvantageous for learners, particularly those with sensory, language or learning impairments. Research and international guidelines show that the ideal ambient level is 30 dBA – 35 dBA, allowing the achievement of an ideal signal-to-noise ratio (SNR) of +15 dB, and the ideal reverberation time is 0.4 s – 0.6 s. Various South African regulations discussed are inconsistent regarding ambient noise level (ranging from 35 dBA – 50 dBA) and say little about reverberation time for classrooms.

**Conclusion:**

South African regulations regarding classroom acoustics require revision to ensure inclusion of all learners with disabilities. The current status does not enforce barrier-free environments in mainstream schools for children with sensory, language or learning impairments.

## Introduction

South Africa (SA) supports a policy of inclusive schooling, striving to accommodate all children, including those with disabilities, in mainstream schools. To this end, the National Department of Education promotes the adoption of barrier-free access to schools for all (Education White Paper 6, 2001). Whether a learner attends a special school for learners with impairments or a mainstream school, barriers to learning should be removed and accommodations should be made to ensure that all learners have the best possible access to education (Department of Education, 2001; World Health Organization [WHO], 2011). The provision of fair, equal and barrier-free education is referred to as inclusive education. According to the WHO (2011), inclusion in this respect means uninhibited access to education for children with special needs, which includes children with disabilities, as well as children who are not necessarily disabled but are disadvantaged in other ways, for example, through race or gender inequality. Thus, inclusive education can broadly be interpreted to mean that all children must have access to education in the least restrictive environment possible, whether in a special or mainstream school.

The design of inclusive environments is a principle that has been adopted by the Department of Basic Education (DBE) (2012) and is applicable in mainstream schools. This approach to design considers the potential ability of all people. It attempts to include as many people as possible, while simultaneously celebrating diversity by recognising the requirement for design solutions that meet the needs of specific types of impairments.

A number of national regulations pertaining to the provision of inclusive schooling exist: The Education White Paper 6 (2001); The National Policy for an Equitable Provision of an Enabling School Physical Teaching and Learning Environment (DBE, 2010); The Guideline Relating to Planning for Public School Infrastructure (DBE, 2012); the Regulations Relating to Minimum Norms and Standards for Public School Infrastructure (2013). These recognise the value of quality special-needs schools while promoting inclusiveness in mainstream education in SA. However, the universal design of the physical environment receives little attention.

A barrier to learning (DBE, 2015) can be anything preventing learners from performing to their full potential. This article focuses on noise as an environmental barrier for learners with language and learning disorders such as dyslexia, dysphasia, attention deficit hyperactivity disorder (ADHD), auditory processing disorder (APD), autism spectrum disorder (ASD) or Asperger Syndrome (AS), as well as those with sensory impairments such as visual or hearing impairments. In addition, children being taught in their second or third language (SAALED, n.d.) are considered, as a significant number of South African learners fall into this category, and it is postulated that this places them at a disadvantage when listening.

In a context of oral teaching and auditory learning, in which 75% of the school day involves listening activities (http://www.asha.org/policy), it is logical that a noisy environment has an impact on learners’ reception of instruction, particularly those with sensory, language or learning disabilities (Nelson & Blaeser, 2010). This is supported by evidence that emerged from the literature.

### Problem statement

Despite the policy to eliminate barriers to learning in mainstream schools in SA, environmental barriers are seldom mentioned. Where environmental interventions are addressed, it is with reference to providing physical access for learners with physical impairments. Insufficient attention is given to other environmental barriers that affect learners with sensory, language or learning impairments. This article specifically investigates the effect of classroom acoustics on these learners.

### Goal and objectives

The goal of this study was to determine whether existing SA norms, standards and regulations regarding classroom acoustics in mainstream schools are adequate to ensure a barrier-free learning environment for all, including those with sensory, language or learning impairments. The following objectives were set:

- to establish the impact that noise has on learners with sensory, language or learning impairments
- to establish the preferred listening conditions for these learners by examining prior research and guidelines available in other countries
- to outline the current South African regulations pertaining to classroom acoustics and assess them against the preferred listening environment.

### Significance

It is established that SA norms, standards and regulations regarding classroom acoustics are inadequate to ensure a barrier-free learning environment for language and learning impaired learners. The implication is that a large percentage of school learners are currently being disadvantaged, and the policy of inclusive education is not being upheld. Children may be performing poorly because of environmental conditions.

## Research method and design

This article provides an overview and evaluation of peer-reviewed, published data concerning noise and learning. It is written as a literature review in a narrative style. The method followed is a systematic review with the aim of identifying, critically evaluating and integrating data with an endeavour to detect gaps or trends and make recommendations for further research. In accordance with Khan, Kunz, Kleijnen and Antes (2003) the systematic review included the following steps:

- Framing questions for review: Background data served to frame the problem statement and established the context for the study. A detailed description of phenomena in question allowed comparisons to be made (Shenton, 2004). Research questions and objectives were subsequently generated.
- Identifying relevant work: A search was conducted on all topics related to the identified problem from the fields of education, audiology, language development and acoustics. Searches on scholarly search engines and academic libraries according to specific keywords and search phrases were conducted. Keywords and phrases included: ‘noise in classrooms’, ‘effect of classroom noise’, ‘inclusive classroom’, ‘reverberation and speech perception’, ‘effect of noise on academic performance’ and ‘classroom accommodation’. The literature study was broad, including both local and international sources.
- Assessing the quality of the studies: Results were assessed according to specified inclusion and exclusion criteria. Where articles were found to reference earlier research, the latter was sought as a primary source. Peer-reviewed articles were included and data from reputable institutions such as universities or relevant professional societies. Articles from blog sites and sponsored by companies in industries related to acoustic products or equipment were excluded. Recent and early literature was considered, with earlier research providing a benchmark for recent research findings. Field studies were given preference over reviews or discussion papers in order to accurately identify the impact of noise on subjects.
- Summarising the evidence: Once relevant data had been sifted, they were organised according to a matrix (Appendix 1). No single paper addressed all the objectives of this discussion at once, thus a meta-analysis of a collection of literature was required. The matrix served to categorise the type of noise, the effect of the noise and the type of learners affected, to identify trends and gaps. National regulations were used to assess the current status of acoustic requirements. International guidelines were examined to establish best practice recommendations.
- Interpreting the findings: Findings were interpreted and critically assessed according to the research questions, while objectives and contradictions in the literature were noted. An objective evaluation of the data was further ensured by the addition of the second author. With each author operating out of a different research field, peer scrutiny was guaranteed (Shenton, 2004).

## Research

The research of literature pertaining to classroom acoustics in the context of inclusive environments was separated according to the three objectives, namely, the impact of noise, ideal listening conditions and the South African regulations.

### The impact of noise on sensory, language and learning impaired children

It is argued that noise presents an environmental barrier to learners with sensory, language and learning impairments, as well as children being taught in their second or third language. The Implementation Report (Department of Education, 2015) presents the results of surveys conducted on the number of the aforementioned learners enrolled in mainstream schools in SA 2013. These results support the WHO’s (2011) statement that close to 20% of learners experience barriers to learning.

The effect of noise and acoustics as a barrier to school performance of children has been a topic of research since the mid-20th century (Yerges & Smith, 1949). However, the effect of noise depends on the variable being measured. When analysing the themes and topics of the literature (Appendix 1), three variables emerged and are present either separately or in combination:

- the effects of different noise conditions on performance
- the effect of noise on the performance of different types of learners
- the effect of noise on different measures of performance.

To meet the objective of establishing whether or not classroom noise and acoustics have a significant effect on learners, each of the identified variables are discussed in detail below.

#### Noise conditions

Noise can be characterised by its loudness, frequency, fluctuation and meaning. Invasive outdoor noise, such as aircraft noise, has been shown to negatively affect learner performance (Haines, Stansfeld, Head & Job, 2002; Hygge, Evans & Bullinger, 2002; Seabi, Cockcroft, Goldschagg & Greyling, 2012, 2015). Studies comparing the performance of children in schools near and distant from airports, and of children in schools near airports before and after decommissioning of the airport bear evidence of this. Noise disturbances of 85 dBA (Hygge et al., 2002) and 95 dBA (Seabi et al., 2012) and equivalent continuous sound pressure levels of around 69 dBA over 24 h characterise the soundscape around these schools and may be considered highly intrusive, periodically interrupting lessons to the extent that teaching needs to be suspended while aircraft pass over.

The loudness of the background noise level implies a decrease in the signal-to-noise ratio (SNR) in the classroom. The SNR is the difference between the sound level of the source sound signal and the ambient (background) sound level (in decibels) and has been shown to be directly proportional to the performance of school children (Jamieson, Kranjc, Yu & Hodgetts, 2004).

Traffic noise has been found to have a similar negative effect on performance, like aircraft noise, as has meaningless irrelevant speech within the class (Hygge, Boman & Enmarker, 2003). Because traffic noise and speech are not as loud and invasive as aircraft noise, it appears that there is something else at play. Evidently, it is not only the SNR in the classroom that produces an effect but rather the characteristic of the noise, in this case, the fluctuation of the noise (Hygge et al., 2003).

Differential performance of school children under different types of background noise supports the notion that both the characteristics of the background noise and the SNR are significant (Klatte, Lachman & Meis, 2010; Dockrell & Shield, 2006). It seems that a significant characteristic of noise that determines the level of disturbance, over and above fluctuation, is the speech content: whether it is present and whether or not it is meaningful.

Furthermore, the listening environment is also influenced by reverberation time. This refers to the amount of time it takes (in seconds) for a signal sound level to die down by 60 dB. The reverberation time is a way of expressing how much a sound reflects or echoes in a room. A long reverberation time has a significant effect on speech intelligibility and perception, especially when in combination with background noise (Klatte et al., 2010; Ljung & Kjellberg, 2009). In a classroom setting, this means that much of what the teacher says in a lesson is not fully comprehended (ASHA Working Group on Classroom Acoustics, 2005).

To conclude, the type of noise in a classroom environment is relevant with reference to its fluctuation, its speech content, the reverberation time and the ambient noise level.

#### Type of learner

While the varying nature of the noise has been shown to have an effect on learners, the type of learner is also a variable that should be considered as they are differentially affected by noise.

The most universal differentiating factor is that of age. A comparison of the effects of background noise and reverberation time on speech perception and listening comprehension of children and adults in a classroom-like setting show that children are more impaired by background noise than adults (Crandell & Smaldino, 1996; Jones, Moore & Amitay, 2015; Neuman, Wroblewski, Hajicek & Rubinstein, 2010). Children only reach adult-like abilities to perceive speech accurately within noise and reverberation as teenagers (Crandell & Smaldino, 2000; Johnson, 2000).

The acoustic conditions of a classroom are particularly important for younger learners. The background noise level (and resultant SNR) has a differential effect even within the age range of 5–8 years, with 7- to 8-year-olds being able to perform better under noise conditions than 5- to 6-year-olds (Jamieson et al., 2004). Children do not have the phonological maturity to reconstruct degraded speech and lack discipline to remain focused on a task in the presence of distracting noise (Edwards & Rogers, 2002; Klatte et al., 2010).

A similar phenomenon can be expected to occur when learners are taught in a language other than their mother tongue. Studies indicate that children taught in their second language are at a disadvantage compared to their peers in the academic environment. The effect of classroom noise on attention and speech perception of second-graders learning in their second language compared to those learning in their first language showed that word recognition in both groups was worse under noise conditions but the effect was greater for second language learners (Nelson, Kohnert, Sabur & Shaw, 2005). English second language learners require a more favourable SNR to ensure good speech perception in the classroom (Crandell & Smaldino, 1996; Peng, 2014). Bilingual learners, who show no difference compared to monolingual controls in word recognition under quiet conditions, perform significantly worse under noise with reverberation conditions (Rogers, Lister, Febo & Besing, 2006).

Apart from and in addition to the inherent handicap of age and the possible disadvantage of language, other types of learners experience exaggerated barriers to learning in the presence of noise: learning or sensory impaired learners. Learners most vulnerable in poor listening conditions are those with hearing impairments (Crandell & Smaldino, 2000). Because of their low hearing threshold, hearing impaired children require the SNR to be at least +10 dB but preferably +20 dB (Finitzo-Hieber, 1981). Reverberation has a significantly worse effect on speech intelligibility for hearing impaired learners compared to normal-hearing learners (Breitsprecher, 2011) and has also been shown to have a worse effect than noise alone. However, the combination of reverberation and background noise has the greatest negative impact on speech clarity (Hazrati & Loizou, 2012).

Speech recognition is influenced not only by the clarity of the auditory signal but also the visual signal clarity (Grant, Walden & Seitz, 1998). From this, it may be deduced that that a visually impaired learner would require increased clarity of the auditory signal to function optimally in the classroom. This is further supported by teaching strategies and guidelines for blind and VI learners, such as the document for student well-being from Newcastle University (n.d.).

A stereotype exists that blind and VI people have ‘super’ senses and can hear better than seeing people (www.dsb.wa.gov). This is not necessarily true. Evidence shows that early blind individuals do not have enhanced spatial hearing and their physical hearing ability is not more developed (Voss, Tabry & Zatorre, 2015). What has been confirmed, however, is that some VI people have a heightened perception of sound and that blind subjects can locate sound sources more accurately than sighted and partially sighted subjects (Lessard, Pare, Lepore & Lassonde, 1998). Rather than being able to hear the teacher better, VI learners are disadvantaged by being unable to follow visual cues and are heavily dependent on auditory communication (Newcastle University, n.d.).

The effect of classroom noise on auditory processing tasks of learning-disabled children, rather than children in general, has been a topic of interest for several decades. An original study by Nober and Nober (1975) found that though learning-disabled subjects performed more poorly than the control subjects, both groups performed worse under the noise condition, demonstrating a universal effect of noise. However, other studies have shown a differential effect of noise on children with learning impairments (Dockrell & Shield, 2006; Ljung, Israelsson & Hygge, 2013).

There is evidence that noise has a significant effect on children with low working memory capacity (Ljung et al., 2013) while some studies considering the performance of ADHD or ASD children compared to typically developing controls show little effect of noise. This may indicate that noise does not affect these types of learners; however, in most such studies, the floor effect is raised as a possible reason for this (Hygge, 2003; Russo, Zecker, Trommer, Chen & Kraus, 2009).

Contrary to these studies, others have shown a positive effect of noise on certain types of learners. Children with ADHD who listened to music performed better in mathematics than controls and subjects in silence or under speech noise conditions (Abikoff, Courtney, Szeibel & Koplewicz, 1996). In another study, learners with attention deficit problems performed better on a memory exercise when exposed to white noise, while controls performed worse under white noise conditions (Soderlund, Sikstrom, Loftesnes & Sonuga-Barke, 2010).

It is thus evident that certain types of learners required different noise conditions to facilitate maximum performance. However, a careful reading of the research reveals that the type of performance being measured is also relevant.

#### Types of performance

The expected performance of school-going children is measured in different areas of development. It has been found that noise does not necessarily have the same effect on each of these areas of performance.

Verbal and non-verbal tasks are differentially affected by different noise conditions. Verbal tasks, such as reading and spelling, are most negatively affected by background conditions of meaningless irrelevant speech, whereas non-verbal tasks are more affected by a background noise condition of environmental noise combined with meaningless irrelevant speech (Dockrell & Shield, 2006). When it comes to memory tasks, recall is more affected by noise conditions than recognition (Hygge, 2003), demonstrating the differential effect of noise on different performance types.

Probably, the most obvious cognitive measure to be affected by noise is that of speech perception and auditory processing, as this relies on the ability to hear clearly. Tasks that rely on hearing, such as interpreting instructions, are negatively affected by a low SNR or long reverberation (Crandell & Smaldino, 1996; Jamieson et al., 2004; Nelson et al., 2005). Speech perception is more disrupted by general classroom background noise, even under low reverberation, while background noise containing speech affects listening comprehension (Klatte et al., 2010).

Apart from not being able to hear, and thus respond to teaching instruction, other cognitive functions have been shown to be influenced by the presence of environmental noise, such as reading comprehension, attention and memory (Hygge et al., 2003; Seabi, Goldschagg & Cockcroft, 2010). Children exposed to aircraft noise, road traffic noise and background speech display a poor reading and comprehension ability (Hygge et al., 2002; Seabi et al., 2012). In some cases where environmental (aircraft) noise was removed, a lag in reading comprehension has been shown to remain (Seabi et al., 2015) but in other cases the reading comprehension effect has disappeared (Hygge et al., 2002). Memory functions have been found to be impaired by noise in the case of aircraft noise, road traffic noise, background speech (Hygge et al., 2002), and white noise (Soderlund et al., 2010).

The preceding review demonstrates noise certainly influences learners and thus poor acoustics proves to be an environmental barrier. The effect of noise is a complex matter because a review of literature has shown that the type of noise, the type of learner and the type of performance being measured are all factors to be considered. No research has been found in which two of these variables have remained constant and only the third tested, e.g. noise condition and type of learner constant with measures of performance tested. However, a meta-analysis of the existing research indicates that all three factors are relevant and should be jointly considered when designing a classroom that is inclusive.

### Preferred listening conditions

The above discussion highlights the relevance of acoustics in classrooms. Although not all learners are equally affected by noise, it may be concluded that on the whole, classroom environments require a low background noise level and a low reverberation time to ensure good speech perception and learner performance.

Although adults are able to understand speech at an SNR of 0 dB, normal-hearing children require an SNR of about +10 dB (Seep, Glosemeyer, Hulce, Linn & Aytar, 2000). In the case of children with hearing impairments, evidence shows that the SNR level should be at least +15 dB (Crandell & Smaldino, 2000). To achieve favourable SNR levels, the background noise levels need to be limited. To this end, most guidelines recommend an ambient level in an unoccupied classroom should be in the region of 30 dBA – 35 dBA.

The American Speech-Language Hearing Association (ASHA) developed thorough guidelines for classroom acoustics for learners with speech-language-hearing disorders, recommending that reverberation time should not exceed 0.4 s, SNR should not be less than +15 dB, and the ambient noise in an unoccupied classroom should be 30 dBA – 35 dBA (ASHA Working Group on Classroom, 2005). It is noteworthy that the specified ambient noise level is for an unoccupied space, as the noise created by occupants is variable and thus does not provide a reliable base for a standard. While classrooms in which hearing impaired learners are to be accommodated should have a reverberation time of close to 0.4 s (Crandell & Smaldino, 2000), the minimum recommended reverberation time for learners being taught in a second language is 0.6 s (Peng, 2014).

Guidelines for acoustic requirements of classrooms from the United Kingdom (UK), the United States of America (USA) and Australia are in agreement regarding the ambient noise level and reverberation times as summarised in Table 1. These guidelines are also suitable for learners with hearing impairments.

**TABLE 1:** Selected international acoustic requirements.

Guideline or standard	Ambient noise level in core learning space (dBA)	Reverberation time in core learning space (seconds)
United Kingdom BB93 (Department for Education, 2015)	35	≤ 0.6
United States of America ANSI/ASA S12.60-2010 (Acoustical Society of America, 2010)	35	0.6
WHO Guideline for community noise (Berglund, Lindvall & Schwella, 1999)	35	0.6
Association of Australian Acoustical Consultants Guideline for Education Facilities Acoustics (Association of Australian Acoustical Consultants, 2010)	35	0.4–0.5

Apart from the ambient noise levels and the reverberation time, some guidelines also refer to the distance between the speaker and the listener. As sound decays over distance, it follows that the further a listener is from the speaker, the weaker the sound signal will be and thus the SNR will be decreased. There is a critical distance beyond which there will be interference between the direct sound field and the reverberant sound (Crandell & Smaldino, 2000). In an average classroom, this critical distance is approximately 3 m – 4 m from the teacher (Crandell & Smaldino, 2000). It is recommended that children with learning impairments should be positioned nearest to the teacher (Klatte et al., 2010).

In summary, to achieve the best listening conditions for all learners, the SNR should be +15 dB, with an ambient unoccupied noise level of 35 dBA, a reverberation time of less than 0.6 s and learners should be within 4 m of the teacher.

### South African policy, regulations and standards

A review of local policies, regulations and standards for acoustic design of classrooms was conducted, which included publications of the DBE and the South African National Standards (SANS).

The National Policy for an Equitable Provision of an Enabling School Physical Teaching and Learning Environment (DBE, 2010) was developed to ensure parity for learners in an enabling learning environment. Recognising that quality education is partly dependent on the learning environment, the policy aims to define and promote an enabling physical teaching and learning environment, including the impact of acoustics on learning outcomes. The policy does not elaborate on design aspects, although it makes provision for the establishment of norms and standards. It is significant that acoustics is recognised as a part of an enabling environment (DBE, 2010, p.27).

The DBE published Guidelines Relating to Planning for Public School Infrastructure in 2012 to ensure inclusive education through set standards for the construction and management of schools, specifying measures for architectural design. It recommends background noise level of 40 dBA – 50 dBA and a reverberation time of 0.6 to 0.7 s. Although the reverberation time recommended is close to that recommended by other international guidelines (Table 1), the ambient noise level is significantly higher than international guidelines and would result in a low SNR in the classroom. It is also not clear whether the ambient noise level refers to an occupied or unoccupied noise level, although unoccupied should be assumed as the occupied noise level is variable, dependent on occupant activities and thus difficult to standardise and measure.

In 2013 the DBE published Regulations Relating to Minimum Norms and Standards for Public School Infrastructure. In line with policy, these regulations call for schools to adhere to principles of inclusive design (Section 6) and comply with requirements of specialised support programmes for learners with special education needs. However, quantitative norms are not given regarding the inclusive design of the physical environment (Section 9). Although Section 18 requires that as far as reasonably possible, acoustics conditions should not impede clear communication or teaching and learning, no guidelines quantify appropriate acoustic conditions.

The SANS 10130 (2008) requires that the unoccupied ambient noise level in a classroom be 35 dBA. This is in line with international guidelines previously mentioned; however, there are no requirements specified in terms of reverberation time. It is further specified that ‘open space’ teaching areas in secondary schools should have an ambient noise level of 40 dBA, while in primary and pre-primary schools it should be 45 dBA. This does not correlate with the research findings that younger learners are more affected by noise than older learners (compare with section: ‘Type of learner’).

SANS 10400 (2010), for the application of the National Building Regulations, does not make provision for acoustic requirements of buildings, explaining in the preface that acoustics is an aspect that only affects comfort or convenience and, being a subjective factor, is difficult to regulate. This is arguable but pertinent to the point that acoustic conditions receive insufficient attention in the design of classrooms.

## Discussion

Inclusive education policies and guidelines are formulated to ensure that learners with disabilities, especially from previously disadvantaged communities, have access to quality education in both special and mainstream schools. The Education White Paper 6 (2001) is largely concerned with teaching and learner accommodations, although inclusive education through inclusive design is also promoted. While it is understood that the policy stemmed from a need to redress historical political exclusions, the guidelines and regulations flowing from the policy do not fully acknowledge the wide range of disabilities found among children of school-going age. Moreover, a lack of detailed attention is given to the role of acoustics in classroom design.

The literature demonstrates the significant impact of classroom acoustics on the performance of learners, particularly those with impairments, who require a lower background noise level and a lower reverberation time to increase speech audibility and clarity. Although it seems that the nature of the background noise is significant, there is general consensus that minimal noise, regardless of character, is preferential.

The case of certain learners performing better on certain types of tasks under certain types of noise is intriguing but not universal. Even within these studies, individual preferences among subjects regarding the noise volume are evident (Soderlund et al., 2010), and such requirements may be best met through the use of personal sound systems.

It is evident from international guidelines, standards and research that classrooms should not be characterised by ambient noise level alone but that reverberation time and even listening distance are also critical aspects. Most international guidelines recommend an ambient noise level of 30 dBA – 35 dBA for unoccupied classrooms. These levels are adequate to accommodate a diversity of learners. The lowest reverberation time to accommodate the maximum number of learners is 0.4 s. This is a very low level, and the majority of literature agrees that a reverberation time of 0.6 s is suitable for most learners.

The recommended ambient noise level of 40 dBA – 50 dBA given in the DBE Guidelines (2012) is not viewed as a satisfactory as it would result in a low SNR. Although the level required by SANS 10103 (2008) of 35 dBA is acceptable, the levels for secondary and primary/pre-primary teaching spaces do not reflect evidence found in the literature that younger learners are more negatively affected by background noise than older learners.

SANS 10103 (2008) does not make any recommendations regarding reverberation time. This is viewed as a shortcoming, as it is clear from the literature that reverberation has a significant impact on the clarity of communication. The recommended reverberation time given in the DBE Guidelines (2012) of 0.6 s – 0.7 s is satisfactory for normal learners but according to the data, it is not ideal for hearing impaired learners and thus falls short of creating an inclusive environment. It is important also to note that the reverberation time in a particular room is largely dependent on the absorption and the volume of the room. This infers that the floor area and ceiling height are relevant as well as the material finishes. Though the DBE Regulations (2013) and Guidelines (2012) are clear on the floor area for classrooms, there is a lack of guidance regarding ceiling height or volume and material finishes.

When the ambient noise cannot be lowered, a suitable SNR can be achieved by amplifying the signal level. This can be done through a frequency modulation (FM) system (DiSarno, Schowalter & Grassa, 2002): a radio transmitter system that amplifies the signal sound, either to a device in the listener’s ear (personal FM) or to room speakers (sound-field FM). However, when using sound-field FM, the overall noise level must be carefully considered with regard to hearing damage or fatigue caused by constant high noise levels. In addition, there is a risk of inter-classroom noise disturbance resulting from the amplified signal being overheard from adjacent classrooms.

## Conclusion

This study undertook a systematic review of the literature on the effect of noise on learning and the ideal listening conditions with reference to the national policy for inclusive education and existing acoustic guidelines, regulations and standards in SA.

It is concluded that SA regulations are inadequate and not on par with international guidelines. Noise is indeed bad for children, particularly those with sensory or language and learning impairments. The ideal ambient level is 30 dBA – 35 dBA, allowing the achievement of an ideal SNR of +15 dB and the ideal reverberation time is 0.4 s – 0.6 s.

These conditions can be achieved by controlling the volume, material finishes and transmission of noise, as well as considering critical distance calculations in classroom design to ensure optimal use of the classroom space for learning.

The following recommendations are made for future research:

- The ambient noise level and reverberation time for classroom design be reviewed and revised in the SA regulations pertaining to classroom acoustics to ensure inclusion of all children with disabilities and to bring them into alignment with each other and with international standards. It is asserted that regulations that accurately describe the ideal listening environment, if diligently applied in practice, will extend the reach of inclusive education.
- The majority of the research reviewed was not conducted in SA. It is recommended that research be conducted locally to determine the extent to which schools in SA comply with local and international standards.
- The literature is silent on the impact of classroom noise on visually impaired learners. It is recommended that a research in this regard be conducted to address this gap.
- That specific research and testing be conducted and design guidelines be established regarding the contribution of layout, materials, finishes and furniture of classroom interior design to optimal acoustic environments for instruction and learning.

## Appendix 1

**TABLE 1-A1:** Literature review matrix

Reference	Guidance				Performance					Noise										Learner impairment/condition								
	RT	SNR	Ambient	Distance	Reading/listening comprehension	Speech perception/ auditory processing	Memory	Maths	Attention	Outdoor			Indoor							Sensory		Learning				Language	Age	No impairment
										Aircraft	Traffic, fluctuating	Traffic, steady	General classroom, no speech	Meaningful irrelevant speech	White noise	Music	Silence	Reverberation	Amplification	Hearing	Visual	ADHD	Dyslexia	SEN	ASD			
Abikoff et al., 1996	-	-	-	-	-	-	-	**x**	-	-	-	-	-	**x**	-	**x**	**x**	-	-	-	-	**x**	-	-	-	-	-	-
ANSI, 2010	**x**	**x**	**x**	-	-	-	-	-	-	-	-	-	-	-	-	-	-	-	-	-	-	-	-	-	-	-	-	-
American Speech-Language-Hearing Association, 2002	**x**	**x**	**x**	-	-	-	-	-	-	-	-	-	-	-	-	-	-	-	-	-	-	-	-	-	-	-	-	-
Association of Australian Acoustical Consultants, 2010	**x**	**x**	**x**	-	-	-	-	-	-	-	-	-	-	-	-	-	-	-	-	-	-	-	-	-	-	-	-	-
Breitsprecher, 2011	**x**	-	-	-	-	**x**	-	-	-	-	-	-	-	-	-	-	-	**x**	-	**x**	-	-	-	-	-	-	-	-
Berglund et al., 1999	-	-	**x**	-	-	-	-	-	-	-	-	-	-	-	-	-	-	-	-	-	-	-	-	-	-	-	-	-
Crandell & Smaldino, 1996	x	-	x	-	x	x	-	-	-	-	-	-	-	-	-	-	-	-	-	-	-	-	-	-	-	-	x	-
Crandell & Smaldino, 2000	-	-	-	-	-	-	-	-	-	-	-	-	-	-	-	-	-	-	-	-	-	-	-	-	-	-	-	-
*Building bulletin 93*, 2015	x		x	-	-	-	-	-	-	-	-	-	-	-	-	-	-	-	-	-	-	-	-	-	-	-	-	-
DiSarno, 2002	-	-	-	-	-	-	-	-	-	-	-	-	-	-	-	-	-	-	x	-	-	-	-	-	-	-	-	-
Dockrell & Shield, 2006	-	-	x	-	-	-	-	-	-	-	-	-	-	x	-	-	-	-	-	-	-	-	-	-	-	-	x	-
Finitzo-Hieber, 1981	-	x	-	-	-	x	-	-	x	-	-	-	-	x	-	-	x	-	-	-	-	-	x	-	-	x	-	-
Grant et al, 1998	-	-	-	-	-	x	-	-	-	-	-	-	-	-	-	-	-	-	-	x	x	-	-	-	-	-	-	-
Haines et al., 2002	-	-	-	-	x	-	-	x		x	-	-	-	-	-	-	-	-	-	-	-	-	-	-	-	-	-	x
Hazrati & Loizou, 2012	x	x	-	-	-	x	-	-	-	-	-	-	-	-	-	-	-	x		x	-	-	-	-	-	-	-	-
Hygge et al., 2002	-	-	-	-	x	-	X	-	-	x	-	-	-	-	-	-	-	-	-	-	-	-	-	-	-	-	-	x
Hygge, 2003	-	x	-	-	-	-	x	-	-	x	x	-	-	x	-	-	-	-	-	-	-	-	-	-	-	-	-	-
Hygge et al., 2003	-	-	-	-	-	-	x	-	-		x	-	-	x	-	-	-	-	-	-	-	-	-	-	-	-	-	x
Jamieson et al., 2004	-	x	-	-	-	x	-	-	-	-	-	-	-		-	-	-	-	-	-	-	-	-	-	-	-	x	-
Johnson, 2000	x	x	-	-	-	x	-	-	-	-	-	-	-	x	-	-	-	-	-	-	-	-	-	-	-	-	x	-
Jones et al., 2015		x	-	-	-	-	-	-	-	-	x	-	-	-	-	-	-	-	-	-	-	-	-	-	-	-	x	-
Klatte et al., 2010	x	-	-	-	x	x	-	-	-	-	-	-	-	x	-	-	-	-	-	-	-	-	-	-	-	-	x	-
Lessard et al., 1998	-	-	-	-	-	-	-	-	-	-	-	-	-	-	-	-	-	-	-	-	x	-	-	-	-	-	-	-
Ljung & Kjellberg, 2009	x	-	-	-	-	x	-	-	-	-	-	-	-	-	-	-	-	-	-	-	-	-	-	-	-	-	-	-
Ljung et al., 2013	-	x	-	-	-	-	x	-	-	-	-	-	-	-	-	-	-	-	-	-	-	-	-	-	-	-	-	-
Nelson et al., 2005	-	-	-	-	-	x	-	-	x	-	-	-	-	-	-	-	-	-	-	-	-	-	-	-	-	x	-	-
Nelson & Blaeser, 2010	-	-	-	-	-	-	-	-	-	-	-	-	-	-	-	-	-	-	-	x	-	x	-	-	-	x	-	-
Neuman et al., 2010	x	x	-	-	-	x	-	-	-	-	-	-	-	-	-	-	-	-	-	-	-	-	-	-	-	-	x	
Nober & Nober, 1975	-	-	x	-	-	x	-	-	-	-	-	-	x	-	-	-	-	-	-	-	-	-	-	x	-	-	-	x
Peng, 2014	-	-	-	-	-	-	-	-	-	-	-	-	-	-	-	-	-	-	-	-	-	-	-	-	-	x	-	-
Robert et al., 2013	-	x	-	-	-	-	x	-	-	-	-	-	-	-	-	-	-	-	-	-	-	-	-	-	-	-	-	-
Rogers et al., 2006	x	x	-	-	-	x	-	-	-	-	-	-	-	-	-	-	-	-	-	-	-	-	-	-	-	x	-	-
Russo et al., 2009	-	-	-	-	-	-	-	-	-	-	-	-	x	-	-	-	-	-	-	-	-	-	-	-	X	-	-	-
Seabi et al., 2010	-	-	-	-	x	-	x	-	x	x	-	-	-	-	-	-	-	-	-	-	-	-	-	-	-	-	-	-
Seabi et al., 2012	-	-	-	-		-	-	-	-	x	-	-	-	-	-	-	-	-	-	-	-	-	-	-	-	x	-	-
Seabi et al., 2015	-	-	-	-	x	-	-	-	-	x	-	-	-	-	-	-	-	-	-	-	-	-	-	-	-	x	-	-
Seep et al., 2000	x	x	-	x		-	-	-	-	-	-	-	-	-	-	-	-	-	-	-	-	-	-	-	-	-	-	-
Soderlund et al., 2010	-	-	-	-	-	-	x	-	-	-	-	-	-	-	x	-	-	-	-	-	-	x	-	-	-	-	-	x
SANS 10103, 2008	-	-	x	-	-	-	-	-	-	-	-	-	-	-		-	-	-	-	-	-	-	-	-	-	-	-	-
Voss et al., 2015	-	-	-	-	-	-	-	-	-	-	-	-	-	-	-	-	-	-	-	-	x	-	-	-	-	-	-	-
Washington State Department of Services for the Blind, 2010	-	-	-	-	-	-	-	-	-	-	-	-	-	-	-	-	-	-	-	-	x	-	-	-	-	-	-	-

RT, reverberation time; SNR, signal-to-noise ratio; ADHD, attention deficit hyperactivity disorder; SEN, special education needs; ASD, autism spectrum disorder.
